# Monthly Meeting of the College of Dentists, London

**Published:** 1859-01

**Authors:** 


### ARTICLE XIII.

### *Monthly Meeting of the College of Dentists, London, June* 1, 1858.

After the usual routine business, Mr. Rymer read the following paper on Mechanical Treatment of Irregularities of the Teeth:—

The subject of the brief observations, which, sir, with the kind indulgence of the meeting, I am about to bring forward, suggested itself to me as one well worthy the attention of the College, from the perusal of a pamphlet on irregularities of the teeth, lately published by Mr. Gaine, of Bath, one of our members.

Mr. Gaine remarks, that it is a rare thing to see a young person with a good set of teeth; by which, I suppose him to mean, a regular *as well* as a healthy set. It is certainly not often that we see *both* these excellent qualities combined, but, as regards the first quality of regularity, although it is common to speak of it as a rare quality, I do not believe it to be so rare as is supposed.

I admit that we seldom see people with *perfectly* regular sets of teeth, but it is an uncommon thing to witness irregularities of so serious a nature as to spoil the appearance of the countenance; such irregularities, nevertheless, *do occur*, and it is one of the most important duties of the dentist to correct them.

The title of this paper is sufficiently explicit as not to lead you to expect me to enter into the subject of the treatment of children's teeth prior to the period when mechanical aid is to be employed, should such aid be necessary. That is a subject which is deserving of separate consideration, as the means of rendering the use of mechanical appliances in many cases *unnecessary*. I therefore come at once to the time, when the temporary teeth are replaced by the permanent, and where irregularities of the latter are fully developed.

The writer to whom I have referred (Mr. Gaine), introduces his views by candidly stating, that whatever novelty may appear in his treatment, the success awarded to his labors has been derived from rightly understanding and correctly applying the principles established by Mr. Fox half-a-century back; he then proceeds to illustrate his method of treatment, by cases which have occurred in his practice.

Case 1 presented this appearance :—Upper jaw—two central incisors and canines projecting ; laterals forced so far within the dental arch, that the lower teeth closed on and over their anterior surface. The two first bicuspid teeth had been removed. The method of procedure is described as follows :—"My first object was to elevate the jaws sufficiently to keep the under teeth from closing on the laterals ; accordingly a gold plate was adapted to *cap* the molar teeth, and secured by means of bands attached to the second bicuspids ; a thick piece of gold was then brought round the front of the mouth, and the plate doubled posteriorly over the laterals—anteriorly over the canines and central incisors, in order to make it sufficiently strong to carry and bear the action of the *screws ;* by this means, I was enabled to keep up a constant, uniform pressure on all the irregular teeth at the same time. In less than a fortnight, the misplaced laterals were made to shut *over* the under teeth, while the projecting centrals and canines were so much improved as to require the insertion of longer screws. In three weeks the cure was complete, and a plate made to cap all the upper teeth. This was worn for six weeks to keep them *in situ* until firm."

I have not quoted the author's exact words, but the actual sense of his first illustrative example has been given in a condensed form for the sake of brevity. Three other cases are mentioned, but as they are examples of precisely the same treatment, namely, pressure applied by means of gold plates and screws, I need not further allude to them.

Now, although this system of practice has been found thus successful by Mr. Gaine, I must say, that I do not like the principle he recommends. I know that gold plates, with and without screws, have been used to correct dental deformities for many years, and are so used still ; but, I believe, all the advantages, and frequently *more* than all the advantages secured in their employment, may be attained by the judicious application of bone and hickory wood ; occasionally, however, a piece, or pieces of gold plate can be used with advantage in *combination* with these materials, but only in such cases as require the teeth to be forced inwards.

I have often seen the most unsatisfactory results of attempts at improvement upon irregular teeth, with ingeniously complicated pieces of metallic mechanism, but I have seldom seen failures where bone and wood have been the agents employed. In their use there need be no complicated machinery, as the following case will show. It is a case somewhat similar to the first example quoted by Mr. Gaine, and will suffice to illustrate the practice to which I give preference. Miss C——'s teeth presented this appearance; upper centrals forced inwards, biting within the lower centrals, the lower laterals inclining posteriorly so that the upper centrals were wedged between these teeth, when the mouth was shut. The upper laterals projected far over the lower teeth and lapped over the adjoining centrals. In the treatment of the case I fitted a bone plate to the upper jaw, covering the grinding surfaces, and cutting edges of all the teeth; the outer, or masticating surface of the plate, being made to meet the bite of the lower teeth. This appliance of course prevented the teeth from closing; just sufficent space was left to allow the irregularities to be acted upon without hindrance from the opposing teeth. I now inserted short box-wood pegs (hickory is preferable,) in the plate immediately behind the central teeth. The dental arch being too contracted to allow of the laterals being placed in their right positions, I also fixed pegs behind the canines and first bicuspid. The plate was then secured in the mouth by means of silk ligatures attached to the second bicuspids. As is always necessary in these cases, I gave particular directions that the plate should on no account be removed. In two days the teeth had been pressed forward to the extent of the length of the pegs. This was, I need hardly observe, the result of the pressure caused by the lower teeth in mastication, etc.; the pegs acting on the inclined-plane principle. The plate was removed and longer pegs substituted for the central teeth ; but the canines and bicuspids did not require these pegs as the small extra space wanted for the laterals was already secured, so that the old pegs were allowed to remain merely in order to prevent the teeth regaining their former places.

In another two days the front teeth had again been forced forward to the extent of the length of the second pegs. New ones were inserted with a similar result in due course, and in ten days the teeth overlapped the lower. Short slips of gold plate were then riveted to the regulating piece just underneath the laterals, and bent outwards and upwards over them. The pressure upon the teeth was kept up laterally (from the central) and inwards, by merely bending the plates with the pliers every two or three days, in the direction the teeth were to take. As before stated these teeth lapped over the central, and it is evident that the pressure just described would push them inwards in a twisted form, but to obviate this, pegs were inserted behind the inner projecting sides of the teeth. In less than a fortnight they had been made to take their proper positions in the dental arch.

The lower teeth were treated upon the same principle as the upper and with like success. Gold plates were worn to keep the teeth in their places until firm, and the case was complete.

In relating the treatment pursued, I have observed, that the dental arch was too contracted to allow the laterals to be placed in their right positions, and that I fixed pegs behind the canines and bicuspids to enlarge the arch. Now in some cases room may be made for irregular front teeth by removing the first bicuspids, but as a general rule I think it bad practice to extract them, although it is a practice too frequently adopted, the evil results of which are especially apparent, where parents have placed their children under the care of those who have not given their whole attention to dental surgery. These teeth should only be removed when the contraction is of such a character as to render anything like a regular arrangement impossible, and even then it is necessary to make sure that the span of the arch will not be so far lessened as to make the patient what is termed "underhung."

It is but recently that we have had a most interesting paper brought before us, on the decay of the first molars, and from the table of operations performed on young persons, for a period of several years, presented with that paper, as well as from our own every-day experience, we learn the great liability of these teeth to decay. It would appear, then, more advisable to remove the first molars, should they be unsound, than the perfect bicuspids. Although I much prefer to save them, if possible, and to avid extraction of permanent teeth altogether.

In what I have said you will notice that it has been assumed that treatment for irregularity commences when all the temporary teeth have been shed. Decided misplacements often occur before this period, and they are more easily overcome, generally speaking; but I make use of the same mechanical appliances to correct them, as in cases undertaken at a later period.

Having thus concisely stated what I consider to be the most efficient as well as the most simple practice as regards the mechanical treatment of irregularity, I wish it to be clearly understood, that efficient and simple though I believe the practice to be, I do not mean to assert, that those who adopt it have no trouble in making crooked teeth straight. They have all the drawbacks occasioned by obstreperous young patients taking out the plates, when at school or at home, which have been carefully adjusted by the dentist—a course which renders the regulation of teeth so vexatious, that few dentists are particularly ambitious of large practice in this department of the profession. As far as I am concerned, I am not now much annoyed in this respect, as I have made it a rule never to undertake a regulation case unless the patient is at home with his relatives or friends. I explain, that unless my directions are attended to in every particular, it will be perfectly useless to proceed with remedial measures. If the plate is afterwards removed, contrary to my wishes, I at once give up the case, unless indeed there appear good grounds for believing the imprudence will not be repeated. By this course, not only is much time saved, which would otherwise have been lost, but one's credit as a practitioner is safe. It is no uncommon thing for good natured dentists to humor their young patients by looking over contined neglect of instructions, and thus they go on and on, perhaps for several months. The natural consequence is, that their attempts at regulation end without any satisfactory results. Parents or guardians get tired of the proceedings, and, as it is scarcely to be expected that people should consider themselves, or those belonging to them, in the wrong, practitioners are condemned as incompetent, although the blame in reality lies with those by whom they are employed.

It has not been my intention, in these imperfect remarks, to enter into the minutiæ of the various contrivances employed in the correction of dental irregularities, but simply to recommend bone plates, and pegs of wood, as preferable in every respect to metallic plates and screws.

I hope the subject will be sifted in the course of discussion, when it is possible my opinions may undergo modification, although I must confess that I have so much faith in the agency for good, of bone and wood, that I scarcely think my mind will be changed. Still, many of us have been led to clearer notions and more correct views on the several subjects which have already been brought before us in papers read from this place, through the very lucid and practical explanations so readily and so kindly given in the course of discussion, by our elder and more experienced members, and if anything should be said this evening in recommendation of a better practice than the one I have adopted and recommended, I hope to be the very first to acknowledge the superiority.

*Special General Meeting*, July 16, 1858.—On Friday evening, July 16th, a special general meeting was held pursuant to a circular issued by order of the council, to revise and amend the laws of the college.

Mr. Peter Matthews, the President, in opening the proceedings, said:—I do not think, gentlemen, that I have many remarks to make in opening the business of this evening, for you all had a circular, with a copy of the proposed amended rules, and are consequently well aware of the purposes for which we are met together. The council consider it requisite that there should be some alteration in certain of the laws, and they have called you together to submit to you the changes which they propose. I shall offer no further observations at present, but will proceed to the business for the transaction of which you have been summoned. (*Hear, hear.*)

Mr. Underwood.—Before any business is gone into, I have to hand in a protest, and I will shortly explain my reasons for doing so. The transactions of the college are known to every member present, and I need not therefore go into them. Those gentlemen who some time ago seceded from the management, had but one intention when they retired from office, and that was so long as the executive which succeeded them should continue to act up to the intentions of the founders, they would not oppose them; they would take no part in the proceedings, but would preserve silence. They would not have interfered with any thing that might have been done here, so long as the executive body acted up to the spirit and intentions of the founders of the college, but they would still have preserved their rights as members of the institution. In the first circular issued after the appointment of the council, it was distinctly stated that no person should be eligible for membership except on examination, and it was upon the faith of that rule that many members of the profession joined the institution, for they felt that if the college were to be of any advantage to the profession—if it were to command the confidence and respect of the public, examination must be the test of admission to the rights of membership and not the mere payment of two guineas a year. (*Hear, hear.*) What I am now saying, proceeds from no captious spirit—I am simply explaining the feeling which many gentlemen besides myself entertain upon this all-important question. The late council used the utmost means in their power to provide for an official examination, and they laid down a *curriculum*. That curriculum however, was not to come into force for two years—the course of examination so laid down was not to commence till October, 1858, so that, had that arrangement been carried out, after the month of October, 1858, no person could have become a member and have received the diploma of the college without a satisfactory examination. Last November the council sent round a statement declaring that they intended to abide by that arrangement, and sixty-four gentlemen joined the college in consequence. But in the month of June, I received a circular from the council announcing that it was not their intention to enforce that by-law. Finding then that the so-called council (*Oh, oh!*) was about to deprive the college of that which I and many other members regard as essential in order to entitle it to the respect of the profession at large, and to the confidence of the public, we considered it our duty to enter our protests in the strongest manner. (*Interruption, and cries of order and chair.*)

A Member.—I rise, sir, to protest against the phrase which has been used—the "so-called" council—there is no "so-called" council. (*More interruption.*)

Mr. Robinson.—I call upon the chairman to put that gentleman down. (*Oh, oh! Interruption and cries of chair.*)

The President.—If you appeal to me, Mr. Robinson, I must do my duty as chairman. (*Hear, hear.*)

Mr. Robinson.—I do appeal to you—I appeal to preserve order.

The President.—Gentlemen, I must appeal to you to support me. (*Hear, hear.*) We have met for a particular purpose, and to that purpose the discussion must be confined. (*Hear, hear.*) I rule that Mr. Underwood is in order, and I must request you to support me in upholding the dignity of the office in which you have placed me.

Mr. Robinson.—I will support you.

The President.—I can hear you, Mr. Robinson.

Another member rose and was about to speak, but was interrupted by cries of "*order*" and "*chair.*"

The President.—I have no other wish than to do my duty; I must deal with all impartially, and in that spirit I have decided that Mr. Underwood is in order. (*Hear.*) I must entreat you who have placed me in my present position to aid me in my endeavor to maintain the honor and dignity of the college, and I will gladly hear what any one has to say, and decide impartially to the best of my ability. (*Cheers.*)

Mr. Underwood.—I assure you, sir, I feel perfectly safe in your hands. Let me say that in using the phrase the "so-called" council, I did it in reference to the paper which I hold in my hand, and not in any invidious spirit. Well, sir, I and my friends who agree with me, felt bound on receiving that circular, to take some step, and we were of opinion that the step we should take was to protest against the council as not having been legally elected. (*Oh, oh!*) It was elected not by law, but in defiance of the law. (*No, no! hear, hear, and order.*) I am not saying this in any invidious spirit, nor with any intention of giving offence, and I claim my right as an Englishman to be heard. (*Cries of order and chair.*)

The President.—I must say, Mr. Underwood, that I think you are now traveling a little out of the way. (*Hear, hear.*) The question is not now whether we are a council, or not; that question has been decided. The council has been elected, and you had as much voice in that election as any other member. (*No.*) You and your friends, you must bear in mind, formed part and parcel of the meeting, and held up your hands for the appointment of the committee which was to select the council from the members, you having declined to remain members of that body. (*Hear, hear.*) I, myself, asked you to consent to be nominated, but you refused. You said—"No, you would take no part in the matter." (*Hear.*) But you voted for the committee, and when the question was put, you held up your hand with the rest. I say, then, that every thing was done openly and according to law. Every man came and voted as he pleased, and no man was prevented. We do therefore, consider ourselves a legally-appointed council; and I must say, that when you talk about protesting against the legality of the council, you are traveling out of the way. (*Cheers.*) The question for our consideration this evening, is as to the alteration of the laws of the college, and if you will confine your observations to that subject, I shall be happy to hear you. (*Hear, hear.*)

Mr. Underwood.—I bow, sir, with all respect, to your decision. I have simply then, as you have so decided, to hand in, on behalf of myself and my friends, sixty-eight protests, and to take my leave of the meeting. At the same time I must request you to see that these protests are duly entered in the records of the college.

The protests were handed to the President, and Mr. Underwood and his friends rose to leave.

The President.—But will you not stay to hear what I have to say in answer to your observations about the examination?

Mr. Robinson.—No sir—we all of us retire.

Mr. Underwood, and the other members who took part with him, then retired from the meeting.

The President.—Now, gentlemen, we will proceed to business. I think you all will agree with me in what I have said, that we are a duly elected council. (*Hear, hear, and cheers.*)

Mr. Purland.—Will you allow me to ask a question of Mr. Tibbs?

The President.—Certainly.

Mr. Purland.—The question has reference to the election of officers. When we met to consider the legality of the late council, Mr. Mackenzie proposed a resolution, which was met by another motion, proposed by Mr. Tibbs, viz., that a committee of five should be elected to fill up the vacancies which had arisen. Upon that committee Mr. Underwood was nominated, together with some of his friends who act with him, but they all declined to serve. My name was then joined with others, to get up a new council; but, before the question was put, I asked Mr. Tibbs—"How will your resolution agree with law No. 4?" His answer was—"I put aside all law: it is to be understood that the laws remain in abeyance until the new council is elected; and I hope, when they are elected, that their first business will be the revision of the laws." No opposition was made to the motion—no word of opposition came from any of the gentlemen who have just left the room; and owing to an observation which I heard, I was induced to look over to where they were sitting; and when the question was put, I distinctly saw them hold up their hands for the appointment of the committee. I would not have sat upon the committee, if I had not been assured that I was acting according to law; and I wish now to ask Mr. Tibbs if I am not right in what I am now saying.

Mr. Tibbs.—I do not quite understand the whole of the question; but the gist of it appears to be—did those gentlemen agree to the appointment of the committee?

Mr. Purland.—And the consequent abeyance of the law, pending the election of the new council?

Mr. Tibbs.—No doubt they agreed as to the appointment of the committee. There can be no doubt in the world about that. And Mr. Underwood himself named Mr. Sass to serve upon it. (*Hear, hear.*) I am not a party man. My sole object is the benefit of the dental profession, and I may honestly say, that I have no personal interest to serve, for I have almost done with the profession, and I have no son who will succeed me.

The President.—Well, gentlemen, these protests have been placed in my hands, but they literally amount to nothing. They protest against the constitution of the council—but we know that we are legally "the council." We have been legally elected by yourselves, and therefore all our proceedings have been legal. (*Cheers.*) I wish, however, that those gentlemen had staid for a few moments; for I should like to have said something to them which perhaps would have done them good. I think, that face to face explanations are always the most satisfactory. There has been so much writing one way, that speaking face to face is really necessary, to enable us to understand each other, and to know each other's intentions. We have found it necessary to revise these laws, and we are going to make a firm stand, to carry out what we have always wished to carry out, the object of the founders of the institution, in making this college a means of raising the status, and upholding the independence of the profession. (*Hear, hear.*) We are not going to abolish the law, but merely to abolish the absurd curriculum which the late council proposed, a curriculum which was more severe than that of the college of surgeons, and which we believe we should find to be impossible in practice. Then the by-law for examining candidates before admission was never confirmed or certified by the members of the college generally; but if our predecessors were so anxious for it, why did they not call a meeting for the purpose, and propose it, and have it confirmed while they were in office? Why did they leave it for the present council to carry out? Upon maturely considering the subject, we felt that we had better confine ourselves to the dental profession. We believe that the arrangements we have made for the dental college of England, are such as will satisfy every unprejudiced mind; for they will, if fairly acted upon, tend to establish the position, and uphold the independence of that profession. I will state briefly the heads of what we propose to do; and I do so in consequence of the statements of those gentlemen who have gone away, that we do not require any examination. This is what we have under our consideration for the future. We intend—these, however, are merely the rough heads, open to such modification as may be thought desirable—we intend, that the law as to examination shall come into operation on the first of January 1861; thereby giving every assistant ample time to perfect himself. We propose then, that every new candidate shall be required to produce a diploma of the Royal College of Surgeons, or some other recognized body, for licensing surgeons. In this we shall be taking a firm and an honorable position. Secondly—We propose that the candidate shall produce a certificate of having been articled to a practicing dentist for a period of not less than four years, which certificate is to be left with the secretary a certain number of days previous to the examination. Thirdly—We propose, that before this law comes into operation, the associates shall be eligible by passing a dental examination only. Fourthly—We propose, that any member, who remains a member three years after the ratification of these rules, shall be entitled to the diploma of the college; and that every member, before these regulations come into force, shall be entitled to his diploma in the third year of his membership. These are the general heads of what we propose. You will see, that every member of the college will be entitled to the diploma after three years of membership, until the new regulation comes into operation, and this, I submit, by giving us three years of supervision, will be, for all present purposes, a sufficient test. I have thought it right to give this explanation, that gentlemen may not misapprehend our intentions. I am most desirous that it should be publicly known, that we do not, as some persons seem to suppose, intend to make this an institution for mere talking, but a college in the strict sense of the term. (*Hear, hear.*) And if the council do not do their duty to your satisfaction, I hope that you will put other persons in their places; but if they do conduct the affairs of the institution efficiently, and to your satisfaction, then I trust you will remember, that they came forward in an emergency to accept the office at your bidding, and to do their best, when none others would undertake the duty. (*Hear, hear.*)

Mr. Tibbs.—I must beg permission to reply to one or two observations that have fallen from the chairman. First with regard to the curriculum which you, sir, have thought fit to characterize as absurd and ridiculous. I was a member of the council by which it was passed, and, ridiculous as it was, every article of it was agreed to unanimously. (*No, from Mr. Perkins.*) I believe, with one exception, it was unanimous. (*No, no.*) I repeat my belief, that it was carried unanimously, except one clause, and that was carried by the chairman's vote. I do not think, therefore, that you can, in justice, to yourself, or to me and those other gentlemen who acted with us, term it a ridiculous curriculum. (*Hear.*) For my part, I felt that it was all that could be desired. You will say, perhaps, more than all that could be desired. I don't think so. I should have been very willing, when I entered the profession, to have submitted to such an examination myself; and I trust you will think again, before you designate it as a ridiculous curriculum. I confess that this is a point upon which I feel very strongly, and I do not think that you are acting wisely in putting off the execution of your scheme for so long a time, because I fear that by doing so, you will lay yourselves open to accusations, that you are blinking the question. In reply to another observation which fell from you, sir, I must remark, that it is not necessary for you to come here to ask the members of the college generally to agree to any by-law that you may make. Your former law, and your intended law says that the council have the power to make by-laws, and there is no condition attached to this power. The council are appointed the executive of the college, and when you have no longer confidence in the council, the proper course is to put them out, and appoint others in their places. If they are to come before the general body to obtain its sanction to every one of their transactions before they are carried into effect, we should be just as well without any council at all. I think that the by-law which you designate as ridiculous, is still the by-law of the college, and I should be sorry to see it departed from in any essential particular.

Mr. Rymer.—I think that Mr. Tibbs is mistaken, as regards the power of the council. The law unquestionably states, that the council may make by-laws, but only such as are in consonance with the fundamental laws of the college. (*Hear, hear.*) Suppose they were to pass a by-law, that the subscriptions should be five guineas a year instead of two; this, I apprehend, could not be carried out without the consent of the general body, because it would be contrary to the fundamental law; and so with reference to this examination, the sixth fundamental law states, that election may take place upon certain conditions, not involving the question of examination. As we exist at present, the council have no power to put their veto on this law, and I think there can be no question as to the propriety of the course we are taking. (*Hear, hear.*)

Mr. Tibbs.—I am sorry to say, that I cannot agree with Mr. Rymer.

A Member.—I submit that that is not the question before the meeting.

The President.—I think it is a material point, and that it is desirable therefore that we should have the opinion of the meeting upon it. (*Hear, hear.*)

A Member.—I quite agree that the law is as Mr. Tibbs has described it, but being somewhat more democratic in my views than many members, I should prefer that all our actions should be submitted to the members at large, and be confirmed by them. That has always been my opinion, but I know that many differ from me; for when I told Mr. Robinson that I felt hurt at what had taken place about the amalgamation question not having been communicated to the members of the college generally, and said, that I thought he might have mentioned the matter to me, seeing that he came to my house day after day to consult about the affairs of the institution, he replied, that it was a question entirely for the council, and with which, I therefore had nothing to do. I said, that I could not agree with him, and that if I were in his place, and he in mine, and upon any subject vitally concerning the members at large, I were asked a question, I should state what had taken place. My opinion is, that every member of the college has as much right to know what is going on in the council room, as the members of the council themselves.

Mr. W. Perkins.—We have in the constitution of the college, a clause, that the council shall have the power to make by-laws. This power is very loosely given, but it never could have been intended that we should have the power to destroy a constitutional law, which we should do, if we were, upon our own authority, to institute a different curriculum to that laid down in law No. 6. If by a by-law we make a different condition for the entrance of members, we in effect destroy that law. And the 17th law says, that no change in the fundamental laws of the college shall be made, except with the consent of a general meeting. Though, therefore, the council have the power to make by-laws, they have not the power to make by-laws that would in effect stultify the constitutional law.

Mr. Tibbs.—But are these same laws to be in force forever?

Mr. Perkins.—No, the 17th law gives you the power of altering them at a special meeting, and this is such a meeting which has been convened for this evening. The council have thought it right to make certain alterations and revisions, and they have called this general meeting to confirm or to nullify what they have done. I thought that the late council was exceeding its power in making the new curriculum, and I opposed every part of it. I was supported in my opposition by Mr. Murphy, of Derby, and one or two others. I opposed the clause altogether, except as regarded that part of it which referred strictly to dental education. All that portion relating to the physiological business, and the clinical business, a slight knowledge of which I consider sufficient for our profession, I opposed. This change, therefore, had not my consent, and I contend, that the council, in passing such a curriculum, abolished the 6th constitutional law, and thereby exceeded their power.

Mr. Tibbs.—What I meant was, that there was no hand held up in the council against it.

Mr. Perkins.—You are mistaken. I did raise my hand against it.

Mr. Tibbs.—Well, you ought to know best.

Mr. Perkins.—Certainly ; for I was the opposing party.

The President.—Now, gentlemen, with your permission we will proceed to the revision of the laws and constitution of the college, and as the proposed changes are submitted to you, I shall be glad to hear any remarks upon them that you may be pleased to make. The first and second laws are not altered; shall we say, therefore, that they are confirmed ?

Laws 1 and 2 were read over and confirmed.

Law 3 was put, and carried as follows:—

"LAW 3.—The college shall consist of members and associates. Honorary members and corresponding members may be elected in accordance with laws 13 and 14."

Mr. Rymer moved law 4.

Mr. Bennett suggested, that an alteration should be inserted, enabling assistants, who had paid their subscriptions, under the idea of one day becoming members, to have the right to vote on questions of vital importance, such as the dissolution of the college.

The President thought, that in such a case, every assistant who had paid his subscription should have the right both to speak and to vote.

Mr. Rymer suggested an amendment to meet the object, and the law was confirmed in the following terms :—

"LAW 4.—The privileges of members shall consist in the right of voting at all general meetings, of attending ordinary monthly meetings, and all lectures and demonstrations which may take place at the college. Each member, whose subscription is not in arrear, shall also be entitled to a yearly copy of the transactions of the college. Honorary members and associates shall have all the privileges of members, but shall have no share in the direction of the affairs of the college."

Mr. Rymer moved, that law 5 be confirmed as follows:

"LAW 5.—The affairs of the college shall be conducted by a president, vice-presidents, a treasurer, a secretary or secretaries, and by a council of fifteen others, to be elected annually, by and from amongst the members of the college. One third of such council shall retire at the end of the year, but shall be eligible for re-election. The president, vice-presidents, treasurer and secretary or secretaries, shall be *ex officio* members of the council. The mode of election shall be as follows:—the chairman and two scrutineers having been appointed, the poll shall commence by each member present personally delivering his balloting paper to the chairman. Country members who have the privilege of voting by proxy, shall, if they exercise their privilege, send their proxy papers under cover, addressed to the chairman of the meeting. The chairman shall deposit all balloting papers in the balloting box. The poll to remain open from half-past seven to nine o'clock in the evening, when the chairman and scrutineers, attended by one of the secretaries, shall retire, cast the poll, and sign the return. In the event of an equality of votes, the chairman shall have the casting vote."

Mr. Jordan asked why assistants were not to be allowed to vote?

Mr. Rymer explained, that in making the distinction recognized in the rule, they were only following in the wake of other similar institutions. There must be a distinction, and it must be recollected, that the assistants were only called upon to pay half the amount of subscription.

Mr. Bennett proposed an amendment, requiring that the names of candidates for the council, nominated by members not connected with that body, should have their names inserted in, and sent out with the house list without distinction, his object being to make the election more popular.

Mr. Thomson seconded the amendment, which, after some discussion, was adopted, and the law was confirmed, with the addition of the following words:—

"Any member of the college shall be eligible as a member of the council, on being proposed by five other members, such proposition to be sent to the council one month previous to the annual general meeting, and the names of such candidates shall be printed with the house list."

Mr. Rymer moved law 6.

"LAW 6.—The subscriptions to the college shall be as follows:—The payment of £21 in one sum to be considered as a life composition; otherwise an annual subscription of £2 : 2s shall be paid by each member. The annual subscription of associates shall be £1 : 1s. Members shall pay a fee of £1 : 1s on entering the college, and associates a fee of 10s 6d.

"All subscriptions shall be due on the first day of January in each year, and shall be payable in advance. Members and associates whose subscriptions are not paid within four months of the date on which they are due, shall forfeit the privileges of membership until the subscription shall be paid. If not paid within twelve months after it is due, the membership shall cease. The council shall, howevet, possess the power to re-admit such defaulting member upon his paying his arrears, if they, in their discretion, think fit so to do, in which case, the entrance fee will not be required."

Mr. Purland.—I propose, that instead of four months, three months be introduced; and that if the money be not paid within twelve months, the defaulting member shall be struck off the list of the college.

A Member.—I second the amendment.

Mr. Perkins.—I think six months would be rather sharp practice. I agree, that the privilege should be withdrawn after three months non-payment, but I think, if the suspended member chooses to come down from his suspension within the twelve months, we should reinstate him on paying the subscription. Remember he will have lost his privileges, possibly for nine months. I admit that we want something definite on this point—we want a law which shall prevent gentlemen who have once been members, coming here and claiming the right to speak, and to interrupt the business of the institution, though they have not paid their subscription, and probably have no intention of doing so.

Mr. Purland.—I have no objection to give way.

A Member.—I think it would be better to let the law stand as it is. A member may be out of the country, and unable to pay his subscription within the time mentioned.

Several Members.—Then let him pay in advance. (*Hear.*)

The President.—We have had instances lately of gentlemen coming to our meetings and making a great noise, interrupting the proceedings, although they had not paid for a long time. I think it hardly fair that persons under such circumstances should be allowed to come in to upset the business, and thwart the wishes of those who have paid their fees. (*Hear, hear.*)

A Member.—Just so ; and if this proposal is adopted we shall have the power to tell some of the gentlemen who were here in the early part of the evening, the next time they come, "you have no right here, and cannot be allowed to stay ;" for I believe I am right in saying as regards several of them, that they have not paid their subscriptions, and that their only object is to break up the college. I say we ought to shut them out.

The President.—I want to shut nobody out ; I want their money. (*Hear, and a laugh.*)

The Member.—But they would shut you out if they could.

Mr. Purland's amendment was then put and carried, and the law as altered was adopted.

Law 7 was agreed to as follows :—

"Law 7.—The treasurer shall demand and receive for the use of the college all moneys due or payable to the college, and shall keep full and particular accounts of all sums so received. An account in the name of the college shall be opened, and all moneys deposited at a banker's, to be appointed by the council."

Mr. Rymer moved the adoption of law 8.

"LAW 8.—Every person desirous of being admitted a member or an associate, must be proposed by two members of the college, the candidate being personally known to one of them, and having deposited his entrance fee, his name shall be placed upon the list of candidates of admission, and their remain at least four weeks previous to the evening on which the ballot takes place, and to secure election, two-thirds of the votes of the members must be in his favor. Should the candidate not be elected, the entrance fee shall be returned."

Mr. Bennett proposed an amendment to the effect that assistants might be nominated by associates, as well as by members.

Mr. Humby seconded the amendment, which after some discussion was put to a show of hands, and negatived. The Law No. 8 was then put and agreed to.

Laws 9, 10, 11, 12, 13 and 14, were severally proposed and adopted.

"LAW 9.—Candidates for membership, on being proposed, shall send, or cause to be sent to the secretaries, or one of them, the following form, properly filled up:

"We, and being members of the College of Dentists of England, recommend of as a fit and proper person to be admitted a member of the college.

"Signed . . . . { ______ ______ }

"Dated

"LAW 10.—The names of new members shall be once advertised in the *Times* newspaper by the council, at the expense of the college, within fourteen days after their election. Such new members shall also be permitted to advertise that they have been elected members, in the local newspapers of their respective places of abode, three times, at their own expense. The following to be the form of advertisement:

"College of Dentists of England.

"Mr. of was duly elected a member of the College of Dentists of England, on the

"Signed

"*Secretary or Secretaries.*"

"Law 11.—No person combining any kind of business with the practice of the dental profession, shall be a member of this college."

Law 12.—No person shall be an associate of the college unless he is an assistant to a dentist. Pupils may be admitted to the monthly meetings, upon the production of an order from the member to whom they are articled."

"Law 13.—Retired members of the profession may be elected honorary members of the college by the council."

"Law 14.—Such members of the profession, residing out of the United Kingdom, as may be considered eligible by the council, shall be nominated corresponding members of the college."

Mr. Rymer moved law 15 as follows:

"Law 15.—No person allowing his name to be associated with disreputable advertisements of any kind, circulating handbills, or exhibiting show cases, boards or placards, shall be a member of this college. And any member making a public exhibition of the diploma, or of any other document granted him by authority of this college, shall have his name removed from the list of members."

Mr. Spencer.—It will be remembered that I endeavored in the council to introduce a law in reference to chemists practicing the dental profession. I am not now, in submitting the question to this meeting, speaking as a member of the council, but one exercising my privilege, simply as a member of the college. We know that we can hardly go through any street in London without seeing written up at every chemist's shop, the word "dentist." We know that these persons are not dentists, and that they can only carry on the dental profession by getting real dentists to work for them, they taking the bulk of the profit, and paying the dentist who does the work, what they please. I will not refer further to the past, because I am sure there is no member of the profession who has worked for a chemist, who does not regret having done so; but I am sorry to say that the evil is growing to such an extent that a very large proportion of the practice is falling into the chemist's hands. I am quite aware that you cannot make a law that chemists shall not be dentists, but you can make a law prohibiting members of this college from working for chemists. It may be objected that you would then offend many members who do work largely for chemists. It may be so; but the same object might be urged against the law prohibiting members of the college from resorting to placards and advertisements. But though there are members who may in times past have resorted to advertising, we find that when they joined the college, they conformed to its rules, and no longer advertised or placarded.

Mr. Thomson.—If dentists would not work for chemists, there would be no chemists practicing as dentists.

Mr. Jordan.—But suppose a chemist is also a surgeon, as many are, why is he not qualified to act as a dentist?

Mr. Tibbs.—Because three irons in the fire are too many to keep hot at the same time. (*Hear.*)

The President.—And I should say he ought to be ashamed of himself to interfere with the practice of another profession. (*Hear, hear.*)

Mr. Jordan.—That may be; but why is he not qualified?

A Member.—Simply because we have a law to say that he is not.

The President.—There are many surgeons who are not qualified to act as dentists.

Mr. Thomson coincided with the President in this opinion.

The President.—The proposition was to exolude all dentists who worked for chemists, because if dentists would not work for chemists there would be no chemists practicing as dentists.

Mr. Jordan.—But you must not suppose that all the working dentists belong to this college; and if they did, it would be rather hard to say that you would deprive a man who might have no other employment, of the means of getting a living by prohibiting his working for a chemist.

The President.—That is not the question. Every working dentist has a right to live, and I should be sorry to say that any man should starve because we prohibit members of this college working for chemists.

After some further discussion, law 15 was postponed for a brief interval, to afford Mr. Spencer an opportunity of proposing an amendment to carry out his views.

Laws 16, 17, 18, 19, 20, 21, 22 and 23, were adopted as follows:—

"Law 16.—The council shall have the power to remove from the list of members, those who, in their judgment, may do anything to the discredit of the college, or the annoyance of its members; but members so removed shall have the right of appeal at a general meeting; such appeal to be made within one calendar month of the date of official notice to the offending member, otherwise the right of appeal will be lost."

"Law 17.—An annual general meeting shall be held in January (at such time and place as the council shall appoint,) to receive the report of the council; to elect officers for the ensuing twelve months, and to transact the general business of the college. Such annual meeting may be adjourned by resolution thereof from time to time, in case the business brought forward should, from any cause, not be concluded."

"Law 18.—The council may, at any time, call a special general meeting: and they shall at all times be bound to do so on the written requisitiou of ten members, specifying the nature of the business to be transacted. Notice of the time and place of such meeting shall be sent to the members at least fourteen days previously, mentioning the subject to be brought forward; and no other business shall be introduced at such meeting."

LAW 19.—At every meeting of the college, or of the council, the resolutions of the majority present shall be binding (except in the case referred to in law 23;) and at such meetings the chairman shall have a casting vote, independently of his vote as a member of the college, or of the council, as the case may be. Members of the college residing beyond twenty miles of Charing Cross, shall be entitled to vote by proxy, for the election of council and officers; and upon all questions at general and special general meetings, upon a poll being demanded in writing, signed by five members. In case of such poll being demanded, it shall be the duty of the chairman to name a convenient day and hour on which the poll shall be opened and closed."

"LAW 20.—The ordinary meetings of the members of the college shall be held on the first Tuesday in each month, excepting in the months of July, August, and September. The business at such meetings shall consist in the election of new members; the exhibition of specimens illustrative of facts and theories in connection with dental science; the reading of papers (the subject being previously approved by the council,) and general discussion bearing upon such papers. The conduct of these meetings to be under the entire control of the council."

"LAW 21.—The council shall have the power to make by-laws; but such by-laws shall not contravene the spirit of the fundamental laws; fill up vacancies in their own number, and in any of the offices, and transact the general business of the college."

"LAW 22.—Every person, upon being admitted a member or associatc of this college, shall sign the following declaration:

'In consideration of my being admitted a member or associate of the college of dentists of England, I hereby undertake to agree and abide by the laws and by-laws of the said college.

'Signed.'"

"LAW 23.—The college shall not be dissolved unless two-thirds of the members shall vote for its dissolution."

The discussion on law 15 was then resumed, and Mr. Spencer moved to insert after the word "placard," the following words by way of amendment—"or acting for, or assisting any person who combines any other business with that of a dentist, shall be a member or associate of this college."

Mr. Perkins.—I am sorry to differ with Mr. Spencer; but by adopting this amendment, I think we should be dealing somewhat too hardly with men, who might be in want of bread. I am as anxious as any one to keep the dental profession in the hands of the dentists, but if a working dentist were actually in want of employment, and a chemist offered it to him, it would be cruel to prevent his accepting it.

Mr. Thomson.—But if the chemist did not get the order, the dentist would. We must recollect, that the chemist has many opportunities of supplanting the profession. A person goes to him to cure the tooth-ache. The chemist says—"You want artificial teeth, I can furnish you with them." He gets the order, and employs the working dentist to execute it, pays him a miserable sum, while he obtains the bulk of the profit. I have recently seen a hand-bill circulated by a chemist, in which he states, that after twelve months' study, he is about to practice the profession of a dentist. He does not say he has been taught, and the probability is that he knows nothing about it. He will get dentists to work for him, and will make a large profit out of their knowledge and labor. It is clear that if the dentists were to refuse to work for him, he could not take the work, and it would then go to the legitimate dentist direct.

Some time ago, I was applied to by a chemist to tell him what books he had best study to qualify himself as a dentist. (*A laugh.*) "But books won't do," said I, "you ought to have some instruction." "That is not necessary," he replied, "I only want to get a little insight into it, that I may practice in a small way." I have myself refused three chemists as pupils: but I consented to take one who intended to practice the dental profession only, and to abandon the chemist's business; and I thought myself justified in so doing.

Mr. Bell.—I entirely approve of Mr. Spencer's amendment, and shall have much pleasure in seconding it.

Mr. Williams.—My opinion accords with that of Mr. Perkins—we are not in a position to put the screw on too tightly. Our object should be to make the college as powerful and useful as possible, but by such a clause we should, I think, exclude many valuable members.

In reply to Mr. Watt.—The President explained, that the word "chemist" was not in the clause; the words were—"who combines any other profession with that of dentist."

Mr. Bennet supported the amendment.

Mr. Tibbs.—As the father of law 8, I beg to offer a few remarks. By-law 8, which I was mainly instrumental in passing, it is declared:—

"That no person combining any kind of business with the practice of the dental profession shall be a member of this college."

Now, this amendment merely brings the assistants under that law. And it ought to be so; for, as has been justly remarked, if there were no dentists to work for the chemists, we should have no chemist-dentists. And I might go still further, and say—if we had had no dentists to work for surgeons, we should have no surgeon dentists. (*Hear, hear.*) You will not be consistent or carry out what you profess—the protection of the public against imposition—unless you make this rule. I say, that we are justified in putting on the screw when we find men trying to pick our pockets. By coming into the profession by a side wind, and making it a collateral branch of their own, these persons can afford to work at a lower price than we can do.

The amendment was, after some further remarks, put and carried, and the law, as amended, was confirmed.

The Chairman.—We now come to the last law.

Mr. Tibbs.—Not yet: I want you to pass another before that. I am told that we are not to pass by-law No. 18, because it is in contravention of some fundamental law. Now, law 2 says—"The objects for which the college is established, are to unite members of the dental profession into a recognized and independent body, and to provide or suggest means of professional education and examination." With that, the by-law in question is perfectly consistent! My proposition is, that the 18th by-law shall be incorporated in the laws of the college. It is—

"Every candidate for membership shall produce certificates satisfactory to the council or body of examiners—

1.—That he is twenty-one years of age.

2.—That he has attended two courses of lectures during two separate sessions on general anatomy and physiology, and special anatomy and physiology of the head, neck, and thorax, and dissected during two separate sessions.

3.—That he has attended two courses of lectures during two separate sessions on general surgery and special surgery of the head, neck, and thorax, with clinical attendance.

4.—That he has attended one course of lectures on therapeutics (including materia medica.)

5.—That he has attended two courses of lectures during two separate sessions on pathology, general and special.

6.—That he has attended two courses of lectures during two separate sessions on the theory and practice of dental surgery.

7.—That he has attended one course of lectures on chemistry and metallurgy, specially applied to dentistry.

8.—That he has attended two courses of lectures during two separate sessions, on the microscopical, histological, and comparative anatomy of the teeth.

9.—That he has attended during two separate sessions, the practical department of the college at some hospital or dispensary connected with the college, or otherwise under some competent professor approved of by the council of this college.

10.—That he has been at least four years studying the mechanical department of dentistry, with some competent dentist, and produce a certificate signed by the said dentist, or his indentures.

11.—The candidate must also, prior to examination, prepare and submit to the body of examiners a written thesis on some subject connected with dental science or art, operate on the natural organs, and also produce to the satisfaction of the examiners, demonstrations of his ability in dental mechanism."

That is what I wish to be incorporated in the laws of the college. I do not expect my proposition to be seconded, but I want it to be entered in the records of the meeting that I have proposed it. I propose it in all soberness and all seriousness; for, I think, that until we have a better one, some such examinations should be undergone by every member. I see no reason why our profession should not stand upon an equal footing with that of the surgeon. I see no reason why the dentist should not attend his patient as a medical man. We have, all of us, constantly cases coming before us that arise from constitutional causes, and can only be cured by medical treatment. But, as it is, the conscientious man must send such cases to the medical practitioner. One half of the cases that we are called upon to deal with are to be cured by medical treatment only. In cases of neuralgia, for instance, you cannot cure the patient by removing the tooth—you merely ease the pain at the expense of the tooth. But, had you the knowledge, which such an examination as I propose would render necessary, you would know how to act in such cases. It is not for me to point out to the council what they should do; but if I was a member of that body, and could not carry out this object, I would not have anything more to do with it. I hope it will be understood that I am speaking in no hostile or invidious spirit, but I think, that these laws having been passed all but unanimously in the council, they should not now be thrown over. I move that by-law No. 18 should be added to the law of the college.

No member having volunteered to second the motion—

Mr. Tibbs said—As the motion is not seconded, it must, of course, fall to the ground; but I beg to protest, and I hope my protest will be duly entered, against this valuable and important by-law being ignored.

Mr. Purland.—It is not ignored; it is only postponed.

Mr. Tibbs.—Then I protest against its postponement beyond the period originally intended; viz. the month of October.

The President.—I admit that there is much matter in your observations, Mr. Tibbs; and as I should be sorry that an opinion should not be taken with respect to your motion, I rise, myself, to second it. In the first place, let me explain that I used a word at the earlier part of the evening which was a *lapsus linguæ;* I did not qualify it by saying, as I ought to have done, that I was speaking of it as the observation made by one of the late members of the council. In the hurry of the moment, having been interrupted by those gentlemen who retired from the room, I did not make that qualification. It was never my intention to describe the curriculum as ridiculous. I ought to have said, that that was the remark made by another gentleman, who, in speaking of it, said—"such a curriculnm for us is ridiculous, and will drive them all to the college of surgeons." It is right I should make this explanation, as I had myself a hand in drawing up this curriculum. Without detaining you further, I beg to second Mr. Tibbs' motion, in order that such remarks as gentlemen may think proper to offer may be made upon it.

Mr. Bell.—This is a question in which I take much interest. The by-law in question lays down a certain curriculum, which, I believe, could never practically be carried out. It says, that candidates for the diploma of this college must, besides undergoing a very strict examination, have regularly attended lectures on anatomy, on pathology, on physiology, and on materia medica, at some one of the medical schools. That is to say, that we should all be surgeons. But I do not see any notice that we are to attend clinical lectures and hospital practice. It is not provided that we are to attend surgery and medical practice, without which, I do not think we should ever be qualified practitioners. Our chairman stated early in the evening, that after the month of January, 1861, every person seeking to become a member of this college must produce a certificate of membership from the college of surgeons or some other recognized surgical body. That with the service of four years under articles to a practicing dentist would, as it appears to me, meet the object in view and give us the status required. But with regard to the examination proposed by Mr. Tibbs, my belief is, that it would be easier, if that were established, to obtain the diploma of the Royal College of Surgeons than the diploma of this college. I think there can be very little question as to which would rank the highest.

Mr. ——.—In reference to one remark made by Mr. Tibbs, I must be allowed to state that this by-law does, in my opinion, contravene the fundamental laws of the college, so far as regards the admission of members.

The question was then put, and there being only three hands held up in its favor, was declared to be lost.

Mr. Tibbs, I thank you, gentlemen, for your votes, for there were two more in my favour than I expected.

The President.—I wish to make one observation as to these protests. The first part of the protest I have already answered ; but, I observe, it goes on to say—"and I further protest against all acts of the council as illegal, and demand that all moneys of the college expended by such council be refunded, each member of the said council being personally liable for the same.'' Now, in regard to this, I can only say that I shall be happy to refund any money which we may have expended in carrying on the business of the college, if it should be the opinion of the majority that we should be held personally liable. (*Hear, hear.*) But the fact is, that we have expended little or no money. All the expenditure has been incurred by the late council; and all that the present council have done in the way of expenditure, has been to pay the debts incurred by their predecessors—the rent of the room, and the servants for their attendance. (*Hear, hear.*) If we were called upon, therefore, to refund what we have expended, it would amount, probably, to not more than a very few shillings a head. As your treasurer, you may depend upon it that I will take care of the funds, and see that no other person shall have it in his power to put his hand upon one farthing of the money that belongs to the College of Dentists of England; and while there are half a dozen members of that college remaining together, it shall be applied for the purposes of the college, and those only. (*Cheers.*) I feel convinced, that upon reflection, those gentlemen who have left us so suddenly this evening, will come forward again to join us to aid in carrying out the objects of the institution for the benefit of the profession at large. (*Hear, hear.*)

Mr. Rymer.—I think it should be distinctly understood, that of the sixty-four gentlemen to whom Mr. Underwood referred as having joined the college in consequence of the circular in reference to the examinations having been sent out, not one of them has made any complaint to the council; but I may add, that if any one of them, or all of them, wish to retire, they may receive back their subscriptions immediately. (*Cheers.*)

The last law was then put and adopted, as follows:—

''No change shall be made in these laws, except at a general meeting of members of the college specially convened for that purpose.''

Mr. Bennett.—We witnessed a most unpleasant scene at the beginning of the evening, when a number of gentlemen retired under peculiar circumstances. Now, it is my belief, that the festering wound which exists amongst the members of the college may be healed, and a healthy feeling restored. (*Hear, hear.*) I would not have presumed to take any step myself in the matter, but that I feel that not having sided with either party (if I may use the expression,) or spoken for or against the one or the other, I may possibly have some influence on that account. (*Hear, hear.*) I therefore beg to submit the following proposition :—

"That a meeting be convened for the purpose of nominating three members of the college who have not taken an active part in the late amalgamation proceedings, to confer as to the best means of bringing about unity in the profession."

I have sanguine hopes, that if three gentlemen who have not taken any active part in the proceedings one way or the other, were to meet and consider the subject, they might devise means for restoring unanimity.

Mr. Tibbs.—I beg to move that the thanks of the meeting be conveyed to the president, for the courtesy, urbanity, and firmness with which he has presided over this meeting. In doing so, I wish to call attention to a remark of the chairman's, in what purports to be a report of the last meeting. I find here that the chairman is reported to have said—"the chairman congratulated the meeting on the result of a communication which he had had with Mr. Tibbs, one of their vice-presidents. He had told him (the chairman) that he would never leave the college, although he had a long way to come. 'I will not desert you,' he said,' 'as others have done.'" Now, I have no recollection of the chairman having said this, or anything like it. If he did say so it was not in my hearing. (*No, no, from the President.*) And I did not like it to go forth to the world uncontradicted that I could animadvert in that way upon the acts of any man or any body of men. I did not say anything of the kind here imputed to me. In my communication with the chairman, I said that I could not accept office, seeing there was no possibility of my being useful in a council which was composed of members whose opinions were at variance with my own on many points. I said that I should be happy to be of service to the council if I could ; but that I could not, at present, see any prospect of being so. As to reflecting upon any one else's desertion, I did not for a moment think of such a thing. Indeed, I know not that any one has deserted. I don't know if the same reporter is taking notes now, but I must say that I was much annoyed at the report altogether—for it is anything but a faithful record of our proceedings. (*Hear, hear.*)

The President.—I quite agree with you, that a more unfaithful report was never written ; and by some accident, the proof sheet was not sent to me as usual for examination, or I should have corrected the errors as far as I could. Unfortunately, Mr. Hughes, our regular reporter—who reports our proceedings admirably—I may say, *verbatim*—was engaged in Parliament, and could not attend. Under those circumstances, I applied to the editor of a paper, and he recommended a gentleman, who, he said, was a reporter engaged on one of the London morning journals, and who, of course, I supposed to be competent ; and you see the result. He put into my mouth what other people said, and what I said he put into other people's mouths. Mr. Hughes, unfortunately, is prevented by the same cause—his Parliamentary duties—from attending here to night ; but we have his *locum tenens*, who, I am assured, is equally qualified. I wrote to Mr. Hughes myself; who, in introducing Mr. Paul —the gentleman who is now taking notes for us—says (alluding to an observation in my letter not to send me such a man as we had last time—one who made a lot of circles and marks of all kinds, which I thought he could not afterwards read,) "I assure you, that Mr. Paul is not merely a man of mark." And now I have no doubt, that with Paul for your reporter, and Peter for your chairman, everything will be done satisfactorily.

The vote of thanks was then put and carried with acclamation, and the meeting separated.

---

*Special General Meeting*, August 7th, 1858.—This meeting was convened in accordance with a requisition signed by ten members, for the purpose of appointing three members who have not taken any active part in the late amalgamation proceedings, to confer upon the best means of bringing about unity in the profession.

The President, in opening the proceedings, observed, that nothing could be more desirable than unanimous and harmonious action in the ranks of the profession; and that it would give him great pleasure to witness a general reconciliation of its members. He was not aware in what manner Mr. Bennett, who had originated the idea contained in the notice convening the meeting, intended to proceed; but he should be happy to hear that gentleman's views, as well as those of others present, on the subject.

Mr. Bennett said, that his object in the present proceedings was to unite the independent members of the profession according to the spirit of the laws of the college. There had been, for some time, every probability of such a result being accomplished, but recent proceedings had rendered it apparent that serious obstacles were in the way. At the last meeting, on the 16th ultimo, was witnessed several gentlemen leaving the room, after handing in a protest. *That* was not the way, in his opinion, of restoring unanimity in the college. But such had been the case, and in order to meet the requirements of the profession, it had occurred to him, that by appointing three perfectly independent members to confer on the best means of putting an end to present differences, there was a greater chance of success than any other way could afford. He begged to move—"that a committee be appoiuted to confer upon the best means of bringing about unity in the profession; such committee to consist of three members of the college, who have not taken an active part in the late amalgamation proceedings; and, that this committee be requested to report thereon to the members at their earliest convenience." (*Hear, hear.*)

The president remarked, that the correct course would be to report to the council whose duty it would be to convene a meeting of the members; when the report would be presented in the customary form.

Mr. Bennett said, he had no objection to leave out the latter clause; upon which—

Mr. Weiss seconded the motion.

Mr. Perkins said it was important that the committee should clearly understand the terms upon which they were appointed. The laws of the college should form the basis of all deliberations, and he considered it should not only be understood, but distinctly expressed, that the committee should report to the council the result of their labors; and he begged to move an addition to Mr. Bennett's motion accordingly.

Mr. Purland could not understand with whom the proposed committee was to confer. He would ask, whether with recusant members or the opposition body?

Mr. Bennett, in reply, intimated that he had framed his motion without any regard to party distinctions. He believed there were twelve hundred dentists in Great Britain, and it was the intention of the college of dentists to unite these into one body. The council being placed in a peculiar position from what had passed, were scarcely, he thought, the proper parties to undertake the task contemplated in his motion, and it was for that reason he preferred that a committee should be nominated; he did not predict that they would be perfectly successful, nevertheless they would collect the opinions of dentists of various ways of thinking, and the result would probably tend to attain the end contemplated in the laws.

Mr. Purland was of opinion that it would be useless to appoint any committee at all. As to the seceders, he did not think their loss was to be regretted. Staunch and firm supporters only were wanted, to carry out their objects, and he believed such composed the present council and body of members. He was glad to find outsiders joining the college in such numbers ; the long list of candidates for admission on the board before them, was sufficient proof that no committee was required as proposed.

Mr. Rymer said he could not agree with what had fallen from his friend, Mr. Purland, as he thought it highly important that general unanimity should be restored, if possible, and he looked upon the proposal of Mr. Bennett as the only means, under existing circumstances, of obtaining so desirable an end. He believed, that whilst two opposing bodies existed, no subtantial benefit would result to the profession. With regard to the several candidates for membership, no doubt it was gratifying that so many were anxious to join the college of dentists, which already was a numerous body ; but even though they numbered eight hundred, and an opposing body of two hundred, or less, was in the field, their efforts would be to a great extent paralyzed ; and so would those of any other society, whether their numbers were large or small, whilst the college continued to carry out its objects independently. The motion of Mr. Bennett would therefore receive his cordial support.

The President did not suppose that there was any intention on the part of Mr. Bennett to reflect on the council, who had been indefatigable in their endeavors to develop the objects of the college, and to protect the interests of the profession generally. He would take the opportunity of informing the meeting what the council had done, in reference to the new medical bill. When that bill was first brought forward, dentists were not even named in any of its clauses. And it did seem to the council, that members of the dental profession should at least be exempted from such of its provisions as affected the recovery of their fees and just remuneration, therefore they placed themselves in communication with several members of the legislature, including Mr. Walpole, upon the subject, and the result was, that dentists were, for the first time, equally exempted from such operations of the act as might prove injurious to them, with the chemists, whose rights had been always respected. It should be remembered, that it was no part of the council's endeavor to be illiberal; they urged that the rights of *all* dentists should be recognized—not of members of the college only. (*Hear, hear.*) Again, it came to the knowledge of the council, that the opponents of the college had been active with Mr. Beresford Hope, in persuading him to insert a clause in the bill, giving the college of surgeons authority to grant special dental diplomas, and to give her majesty power to grant a charter of incorporation to a "British College of Dental Surgeons." Thus the opposing party would have copied those who have been at so much pains to rear the college of dentists, and even endeavor to deprive them of their name. But Mr. Hope was immediately waited upon—the constitution and objects of the college of dentists of England was clearly explained to him, and he at once decided upon striking out the latter and offensive portion of the clause. He mentioned these things to show that the council had not been inactive, and it was necessary that the members should know that they were not neglectful of the interests of the profession, as attempts had been made to overthrow the college by false reports, and endeavors to damnify their proceedings. Matter had appeared in print which was most unjustifiable, and which, it was to be feared, emanated from some of those who aspired to be looked upon as the more enlightened portion of the profession. (*Cheers.*)

Mr. Bennett had no intention whatever to reflect upon the council , if they felt themselves in a position to effect the object of his motion, he would say, let them do it ; but he was still of opinion, that it would be best accomplished by a committee of three independent members. (*Hear, hear.*)

Mr. Bell quite agreed in the propriety of appointing the committee, but thought three too small a number to undertake such arduous duties.

Mr. Rymer said, that power might be given the committee to add to their number; he feared a difficulty might be experienced in obtaining the services of even three gentlemen for so delicate a duty as that contemplated in the motion.

The motion was then put from the chair, with the latter clause omitted, and the following words substituted—"and that such committe report to the council at their earliest convenience."

Carried *nem. con.*

The following gentlemen were then duly proposed to act on the committee, and unanimously approved:—Mr. S. Tibbs, Cheltenham; Mr. W. Bennett, Berners street; Mr. Owen, Islington.

A vote of thanks having been accorded to the President, for his conduct in the chair, the proceedings terminated.

---

*Special General Meeting, August* 25, 1858.—A special general meeting of the members was held on Wednesday, the 25th of August, at the rooms of the association, in Cavendish square, for the consideration, and confirmation if approved, of the regulations adopted by the council, relative to candidates for the diploma of the college.

The President in the chair.

The President opened the business by observing, that the proceedings of the college, from the very commencement, had been entirely public. There had been no closed doors, except at the council meetings; and every one had had an opportunity of hearing their discussions. They had permitted reporters to attend, and to take notes of all that was said; but he was sorry to find, that this privilege, so freely accorded to all, had been somewhat abused; and seeing the reporter of the "British Journal of Dental Science" present, he should take the liberty of asking him, whether the report of the last meeting had been written out by him; because it was so imperfectly rendered that he did not think that any gentleman connected with the press could allow such a report to be published? They knew how truthfully and accurately their own reporter always represented their proceedings; and although he (the president) should not always require an absolutely verbatim report, he should stand by the maxim of Lord Denman, that if the reporter of a journal gave a "garbled" report of the proceedings, he was liable to an action at law. Lord Denman said clearly enough, that you need not publish every word that was said; but that your report must not be a garbled state ment. Now their president, unfortunately, had been placed in this very sad position, that he had been made to say, in the "Monthly Journal of Dental Science," things which he had never uttered; whilst what he had said had been struck out; so that the whole pith and scope of his argument had been lost. (*Cheers.*) In fact, his speeches had been so garbled, that he did not recognize the version of his own utterances. As the reporter for that periodical was present, he thought it was fair to that gentleman, as well as to himself, that he should ask whether the report, as published, was the report which he had furnished to that paper?

The reporter declined to answer.

The President said, as he could elicit no response, he hoped the reporter, in future, would endeavor to represent fairly what occurred at their meetings; that he would not strike out whole sentences, which were very important, in order to insert mere verbiage, which was not of the slightest importance. Passing from this, however, and coming to the actual business of the meeting, he had to submit to them the names of numerous gentlemen who were proposed as members, and with respect to whose election he had to take their "yea" or "nay."

The following names were then read over, and every gentleman proposed was unanimously elected:

E. Carter, Esq., Barnsley, Yorkshire; T. P. Fay, Esq., Liverpool; E. Hargreaves, Esq., Manchester; J. Horne,

Esq., Glasgow; T. K. Law, M. D., D. D. S., Santiago, Chili; G. Lydden, Esq., (Associate,) Woburn Place, London; W. Margetson, Esq., Dewsbury, Yorkshire; Donald Macleod, Esq., North Shields; B. L. Mosely, Esq., Hull, Yorkshire; H. Shephard, Esq., Southsea, Portsmouth.

Mr. Rymer then read the circular convening the meeting.

The President said that they had now heard the curriculum which was prepared by the council as the condition of membership, and he trusted that they would approve of it. He regretted that there were not more members of the college present that evening, and reasons no doubt could be assigned for their absence. He did not like, however, to put reasons into any man's mouth; but he thought the absence of a constituency might generally be regarded as a proof of confidence in the representatives. (*Cheers.*) Without further observations, he should call upon Mr. Bell, the original propounder of those resolutions, to submit them to the meeting.

Mr. Bell said that, as there were so few members present, it was not necessary for him to enter upon a minute discussion of the merits of the resolutions. They had been fully argued and canvassed by the council; and he was happy to say that they had been approved by that body. However, as the meeting was a small one, and as he had a very great desire that resolutions of this importance should be submitted to the opinion of every member of the college, he should beg to propose that the views of the members at large should be taken upon them by means of proxy. (*Cheers.*)

Mr. Rymer suggested that the correct business course would be for Mr. Bell to move the resolutions at that meeting, and to submit them for approval to the general body afterwards. Of course they were aware that the majority of their members resided many miles away from London; and he recommended, therefore, that these matters, all important as they were, should be decided by a general poll.

The President thought it important that the recommendations proceeding from that meeting should be fully endorsed by them; because country members naturally looked to what the London men said; and if they found resolutions unanimously carried in London, they would most probably approve of them.

Mr. Bell then formally moved the first resolution.

"Candidates shall be required to produce the diploma of the 'Royal College of Surgeons,' or that of some recognized home or colonial licensing surgical body."

Mr. Humby seconded it, and expressed his conviction that there could be no two opinions with respect to such a proposition.

Mr. Rymer supported the motion. It was important in proposing a resolution of this nature, that they should vindicate themselves from any charge of inconsistency, which might possibly be brought against them. They were a perfectly independent body, and yet they spoke in that resolution, of going to the college of surgeons for a diploma. His view had been in favor of complete independence; and as the resolution was now proposed, it would be seen that they were not compelled to go to the Royal College of Surgeons, but to *some* recognized licensing body. The licensing surgical bodies were established corporations. If there were no such institutions, he apprehended that the dentists would very soon originate one for themselves; and he did not see, therefore, that by this resolution they made themselves at all dependent upon the college of surgeons. If they had declared that they must go before the college of surgeons, and be examined by a mixed board of dentists and surgeons, they would be in fact tacked on to the tail of the college. But the difference between that proposition and the resolution as it stood, was clearly manifest; and he thought, besides, that it was a perfectly liberal proposition, for it was not to come into operation before three years.

Every respectable dentist would have the opportunity, in the interim, of becoming a member of the college, and if he did not avail himself of it, he would have no just ground of complaint. (*Cheers.*) He, therefore, supported the resolution, fully believing that it would place the college of dentists in the position which they deserved to occupy. He was satisfied that, ultimately, they would become a united body. (*Cheers.*)

The President wished to supplement a very few words. It was well known that he personally had no objection whatever to go before the college of surgeons. So far from it, he had, when a young man, entered at St. George's Hospital, and had gone through all those preliminaries which were essential to becoming a surgeon; but a very serious domestic calamity, the loss of his eldest daughter, just as he had terminated his studies, quite prostrated him, and compelled him to forego that which was, at the time, a very ardent wish. Desiring now that they should be quite independent, he thought that there was nothing in the resolution which should excite the jealousy of the most sensitive; and he believed that its effect would be to give them a *starting*, and to place them considerably in advance of their present position.

The resolution was then put to the meeting, and it was carried unanimously.

Mr. Bell proposed the following resolution:—

"Candidates shall also be required to produce a certificate of having been articled to a dental practitioner for a period of not less than four years. Such certificate to be delivered to the secretary of the college, thirty days before the candidates shall be eligible for their dental examination."

He had, he said, much pleasure in introducing it to their notice, but as it had been carefully considered and discussed, and as his name was appended to it, it was not necessary that he should occupy their time by telling them that he approved of the details of the proposition.

Mr. Stoddart seconded the motion, merely observing, that it was consequent upon the first.

It was carried unanimously.

Mr. Bell proposed, and Mr. Imrie, jun., seconded the next resolution.

"Associates of the college enrolled, and pupils who shall have been articled to members of the college, previous to the foregoing regulations coming into operation, shall be eligible for membership, by passing a dental examination only.

Mr. Bell proposed, and Mr. Weiss seconded the last resolution :—

"Any member who shall maintain his membership for a period of three years after the ratification of these rules, shall be entitled to a diploma of the college. And any person who may become a member previous to the foregoing regulations coming into force, shall be entitled to the diploma of the college upon the expiration of the third year of his membership."

Both were carried unanimously.

The President said, he trusted he should be excused, if he expressed the pleasure he felt at the unanimity with which they had passed the resolutions. He was satisfied that they ought to stand or fall by their college; because he believed, that as established, it would help to maintain their position, and would in no way derogate from their dignity. (*Cheers.*) Still, these resolutions were so important, that he thought they ought to test the opinion of the members by a poll; and he hoped, therefore, that that suggestion would be acquiesced in. He had addressed meetings of very great consequence, and where the disposal of large sums of money was at stake, at which there were not so many gentlemen present as there were at that moment; but as had been before observed, the absence of members only proved their confidence in the executive. (*Cheers.*)

Mr. Rymer then demanded a friendly poll.

The Chairman said he quite agreed in the propriety of a poll; because he wished that the opinion of every member of the college should be taken, either personally or by proxy. He should appoint the poll to be taken on Wednesday, 22d September, at eight o'clock.

Mr. Rymer observed, that that was the anniversary of their first meeting, two years ago, at the London Tavern. He also remarked upon the inaccurate statement contained in a letter addressed to the editor of the "British Journal of Dental Science," with respect to the numbers present at their last meeting. Instead of their being only fifteen members present, after the dissentients had left the room, there were thirty-nine. This he could say positively, for he happened to count the numbers. (*Cheers.*)

Mr. Purland, in a courteous and complimentary speech, proposed a vote of thanks to the President for his able conduct in the chair.

The President acknowledged the compliment. He reminded the meeting that he was their Treasurer, as well as their President, and that he only occupied the latter position until they should elect some other person to take his place. He was there, however, by the favor of their good opinion, and he relied upon the continuance of that support which had invariably been extended to him.

The proceedings then terminated.

[*Lond. Quar. Jour. Dent. Sci.*

